# Mechanisms underlying CSD initiation implicated by genetic mouse models of migraine

**DOI:** 10.1186/s10194-025-01948-x

**Published:** 2025-01-27

**Authors:** Daniela Pietrobon, K.C. Brennan

**Affiliations:** 1 Department of Biomedical Sciences and Padova Neuroscience Center, University of Padova, Via Ugo Bassi 58, 35131 Padua, Italy; 2 Department of Neurology, University of Utah, 383 Colorow Drive, Salt Lake City, UT 84108 USA

**Keywords:** Spreading depolarization, Familial hemiplegic migraine, Migraine with aura, Glutamate, NMDA receptor, Ca_V_2.1 channel, Na_V_1.1 channel, α2 Na/K ATPase, Casein kinase 1δ

## Abstract

A key unanswered question in migraine neurobiology concerns the mechanisms that make the brain of migraineurs susceptible to cortical spreading depression (CSD, a spreading depolarization that underlies migraine aura and may trigger the migraine pain mechanisms). Important insights into this question can be obtained by studying the mechanisms of facilitation of CSD initiation in genetic mouse models of the disease. These models, all generated from families with hereditary migraine, allow the investigation of the functional consequences of disease-causing mutations at the molecular, cellular, synaptic and neural circuit levels. In this review, after describing the available genetic mouse models of migraine, which all share increased susceptibility to experimentally induced CSD, we will discuss the functional alterations in their cerebral cortex and the mechanisms underlying the facilitation of CSD initiation in their cortex, as well as the insights that these mechanisms may give into the mechanisms of initiation of spontaneous CSDs in migraine.

## Introduction

Cortical spreading depression (CSD) is a slowly propagating self-sustaining wave of nearly complete depolarization of a sizable population of brain cells that lasts about one minute and silences brain electrical activity for several minutes (hence the name spreading depression) [1–4]. There is strong evidence from human and animal studies that CSD is the neurophysiological correlate of migraine aura and also a trigger of migraine pain mechanisms ( [3, 5–7] and references therein) [8–10]. A key unanswered question in migraine neurobiology concerns the mechanisms that make the brain of migraineurs susceptible to CSD.

Understanding the initiation of CSD in the normally metabolizing, apparently uninjured brain of a migraine subject is a nontrivial proposition. In experimental models, it requires intense stimuli, which depolarize a minimum critical volume of brain tissue, increase the extracellular concentration of potassium ions [K^+^]_e_, and release glutamate (and other neurotransmitters) [2, 3] (Box 1). Already the first studies of CSD mechanisms in the 50s pointed to [K^+^]_e_ ( [11] and/or [glutamate] [12] as key players in CSD initiation. Both experimental data and computational models support the idea that increases of [K^+^]_e_ and/or extracellular glutamate above critical values are key events for CSD ignition [2, 3, 13–16].

Important insights into what makes the brain of migraineurs susceptible to CSD can be obtained by studying experimentally-induced CSD and the mechanisms underlying facilitation of CSD initiation in genetic mouse models of the disease. These models, all generated from families with hereditary migraine, allow the investigation of the functional consequences of disease-causing mutations at the molecular, cellular, synaptic and neural circuit levels. In this review, after describing the available genetic mouse models of migraine, which all share increased susceptibility to experimentally induced CSD, we will discuss the functional alterations in their cerebral cortex and the mechanisms underlying the facilitation of CSD initiation, as well as the insights that these mechanisms may give into initiation of spontaneous CSDs in migraine.

### Box 1 Minimum conditions for CSD induction, examined in wild type animals

There are two studies that systematically examined the induction of CSD, using K^+^ as the initiating stimulus, in uninjured tissue. Matsuura and Bures [17] extrapolated the minimum volume of cortical tissue in anesthetized rats by injecting K^+^ from two electrodes at varying distances. Tang et al. [14] used a microfluidic device with mouse brain slices to expose the cortex to different concentrations of K^+^ over different surface areas. Matsuura and Bures reported minimum tissue volumes required for CSD induction between 0.9 and 3.7 mm^3^. In contrast Tang et al. reported volumes an order of magnitude smaller – between 0.03 and 0.06 mm^3^. The studies were very different and each has advantages and disadvantages, but they both contribute to our understanding. Matsuura and Bures used large (1 mm diameter) capillaries, inserted 1 mm into the cortex, to induce CSD, and did so repetitively. The cortical injury caused by this technique likely reduced overall CSD susceptibility. Moreover, they also used supraphysiologic concentrations of K^+^ throughout (minimum 1.5% KCl solution; 201 mM). However, Matsuura and Bures clearly established that CSD induction involved both spatial and temporal summation. Expressed in terms relevant to uninjured cortex, this means that two (or more) foci of subthreshold K^+^ elevation could combine, either via spatial proximity or repetition in time, to generate a CSD event.

Tang et al. [14] focused exclusively on (patho)physiological K^+^ concentrations, with a maximum of 145 mM, the intracellular concentration likely relevant to tissue injury. They found that there was an inverse relationship between [K^+^]_e_ and surface area exposed to elevated K^+^ that was relatively constant between 15 and 145 mM, meaning that higher [K^+^]_e_ were associated with lower surface area (and thus volume) of tissue needed to induce CSD, and vice versa. At and below 15 mM (at approximately the ‘K^+^ ceiling’ of 12 mM established by Heinemann and Lux [18]) the relationship flattened significantly, with only whole-slice perfusion sufficient to induce CSD.

There are several implications to this work. The first is that the closer one approaches the limits of normal K^+^ physiology (at which K^+^ is able to be actively buffered) quite large areas are needed to achieve CSD ignition. This would appear to suggest that fairly widespread disruption of cortical excitation/inhibition balance would be needed for CSD ignition in migraine. However, at higher [K^+^]_e_, much smaller areas and volumes were needed. While these ranges appear well beyond the normal physiological range, they need not be ruled out in migraine pathogenesis under two conditions: that the insult is recoverable without damage, and/or that the region affected is smaller than can be detected with current technology. It is possible that both of these conditions can be met: the cortex is known to recover from CSD itself, which involves [K^+^]_e_ elevations to 40–60 mM [3]. Moreover, the tissue depolarization volumes modelled by Tang et al. [14] approximate the volume of a cortical column or the perfusion volume of a penetrating arteriole [14, 19, 20]. These volumes are smaller than the resolution of conventional MRI in humans, so any damage caused by such events might elude detection currently. These volume factors are also hypothesis-generating regarding possible mechanisms: it may be easier to envision a single cortical column undergoing a significant supply–demand mismatch or excitation/inhibition imbalance than a larger region; and/or a transient disruption of perfusion in a penetrating arteriole, whether due to brief thrombosis/thrombolysis, or local vasospasm.

The studies, discussed here [14, 17], suggest that there are two potential paths to CSD ignition in the uninjured conditions we assume prevail in migraine: a wide region experiencing a modest elevation in [K^+^]_e_, ~ 12–15 mM; and a potentially much smaller region experiencing larger (but still physiologically plausible) elevations, > 40 mM. That said, we suspect that CSD triggering in non-injury conditions is mostly, if not always, multifactorial. One likely contributing factor is metabolic compromise. At the 12–15 mM [K^+^]_e_ tipping point, where normal cortical physiology turns toward the excitable attractor state of CSD, the contractility of perfusing arterioles reverses. Arterioles dilate below ~ 12–20 mM [K^+^]_e_, due to hyperpolarization by inward rectifier K^+^ channels, but beyond this concentration, these regulated mechanisms fail and arterioles constrict due to direct membrane depolarization [21, 22]. Thus, at the exact point where increased perfusion could help rescue neurons in the irrigated region from further depolarization, perfusion decreases.

## Genetic mouse models of migraine

Thus far there are eight monogenic migraine mutations that have mouse lines associated with them, allowing investigation of the underlying mechanisms. Seven of these are derived from patients with familial hemiplegic migraine (FHM, a rare subtype of migraine with aura) and one from patients with both migraine with aura (MA) and familial advanced sleep phase syndrome (FASPS, a rare sleep condition in which individuals go to sleep unusually early in the evening and wake early in the morning). Apart from the motor weakness or hemiplegia during aura and the possible longer duration of the aura, typical FHM attacks (as those occurring in pure FHM) resemble the attacks of common MA, and both types of attacks may alternate in patients and co-occur within families. FHM and MA are considered to be part of a clinical and genetic spectrum [23–26]. However, some FHM mutations may cause “atypical” severe attacks and additional ictal and/or permanent neurological features such as epilepsy, loss of consciousness, ataxia and cognitive impairment [23, 24, 27].

FHM type 1 (FHM1) is caused by missense mutations in the gene CACNA1A, encoding the pore-forming subunit of the voltage-gated calcium channel Ca_V_2.1 [28]. The Ca_V_2.1 (P/Q-type calcium) channel is widely expressed in the nervous system, including all structures implicated in the pathogenesis of migraine, and plays a dominant role in controlling neurotransmitter release, particularly at excitatory and inhibitory central synapses ( [29] and references therein). In addition to its presynaptic localization, additional somatodendritic localization of Ca_V_2.1 points to postsynaptic roles, e.g. in regulation of neuronal intrinsic excitability and gene transcription [29].

FHM type 2 (FHM2) is caused by mainly missense mutations in the gene ATP1A2, encoding the α2 Na/K ATPase (α_2_ NKA) [30]. The α_2_ NKA is expressed primarily in neurons during embryonic development and at the time of birth, and almost exclusively in astrocytes in the adult brain [31–36]. At cortical excitatory synapses, the α2 NKA is colocalized with the glutamate transporters GLT-1 and GLAST at perisynaptic astrocytic leaflets [31, 34, 37], where a large fraction of GLT-1/α_2_ NKA couples exhibit a separation distance indicative of physical coupling [34]. In contrast, the α_2_ NKA is not present in the large majority of astrocytic processes surrounding inhibitory synapses [31, 34]. The α_2_ NKA, which is the only NKA pump significantly expressed in cortical astrocytes [34, 36, 38], plays a key role in K^+^ and glutamate clearance during neuronal activity [37, 39, 40] ( [41] for review and references). Moreover, being essential for several astrocytic transporters relying on Na^+^ influx, its function is also important for astrocytic Na^+^ homeostasis, Ca^2+^ homeostasis and pH regulation [41–44].

FHM type 3 (FHM3) is caused by missense mutations in the gene SCN1A, encoding the pore-forming subunit of the voltage-gated sodium channel Na_V_1.1 [45]. The Na_V_1.1 channel is highly expressed in inhibitory interneurons in several brain areas; it is mainly localized at the axon initial segment and plays a key role in interneuron excitability, particularly in sustaining high-frequency firing [46–48]. Indeed loss-of-function mutations in the Na_V_1.1 channel cause a spectrum of epilepsy syndromes [49].

In two families exhibiting MA and FASPS, both neurological phenotypes segregated with mutations in the casein kinase 1δ (CK1δ) gene [50]. CKIδ is a ubiquitous serine-threonine kinase that phosphorylates the circadian clock protein Per2 and many other proteins involved in brain signaling [51–54].

Knockin mouse models for FHM1, FHM2, FHM3 and MA/FASPS were generated by introducing human mutations in the orthologous genes using homologous recombination [55–60] or by inserting human mutations into the mouse genome via bacterial artificial chromosome technique [50, 61, 62]. Four of the FHM mouse models carry mutations which cause in humans typical FHM attacks without additional clinical features (pure FHM): the R192Q mutation in the Ca_V_2.1 channel [55], the W887R mutation and the E700K mutation in the α_2_ Na/K ATPase [57, 61] and the L1649Q mutation in the Na_V_1.1 channel [60]. Two knockin mouse models carry mutations (S218L in the Ca_V_2.1 channel [56] and G301R in the α_2_ Na/K ATPase [58]) which cause more severe FHM attacks that may include, in addition to hemiplegic migraine, prolonged coma/torpor or confusional state, epileptic seizures, elevated temperature, cerebral edema, and may also cause transient or permanent cerebellar signs such as ataxia, nystagmus and cerebellar atrophy [63, 64]. Another knockin FHM mouse model carries the L263V mutation in the NaV1.1 channel [59], which was identified in a family in which independent attacks of epilepsy co-occurred with hemiplegic migraine in three of five mutations carriers [65]. The MA/FASPS genetic mouse model carries the CKIδ T44A mutation, one of two mutations that were identified in two families with phenotypically normal MA (not involving hemiplegia) [50]. The T44A mutation reduces the catalytic rate of the enzyme in vitro consistent with a loss of function in CKIδ [50, 66].

Both the R192Q and S218L FHM1 mutations produce gain of function of recombinant human Ca_V_2.1 channels, mainly due to increased single channel open probability and channel activation at lower voltages [67–69]. The gain of function effects of the S218L mutation are larger compared to those of the R192Q mutation, in correlation with the more severe clinical phenotype caused by S218L. A gain-of-function effect on human recombinant Ca_V_2.1 channels was also found for other 5 FHM1 mutations analysed at the single channel level [29, 70]. Accordingly, the genetic mouse models of FHM1 show an increased Ca_V_2.1 calcium current in different types of excitatory neurons, including cortical pyramidal cells [29, 55, 56, 71–74]. In agreement with the lower activation threshold of human S218L Ca_V_2.1 channels compared to R192Q Ca_V_2.1 channels [69], the gain-of-function of the P/Q-type calcium current was larger in neurons of S218L compared to R192Q FHM1 mice [55, 56]. As a consequence, in excitatory synaptic terminals, a fraction of mutant Ca_V_2.1 channels was open at resting potential in S218L (but not in R192Q) FHM1 mice [73, 75]. The gain-of function effect of the FHM1 mutations may be neuron type specific, as shown by the unaltered Ca_V_2.1 current measured in cortical inhibitory interneurons [76] and capsaicin-sensitive trigeminal ganglion neurons [72] in the R192Q FHM1 mouse model. This probably reflects the fact that the gain-of-function effect may be dependent on the specific Ca_V_2.1 splice variants and/or auxiliary subunits [77, 78].

Both the W887R and G301R FHM2 mutations produce complete loss-of-function and impaired membrane targeting of human recombinant α_2_ NKAs expressed in human cell lines [30, 57, 79, 80]. Accordingly, the brain of heterozygous knockin mice carrying either the W887R or the G301R FHM2 mutation show an about 50% reduction in the expression of the α_2_ NKA [57, 58]. In contrast, the E700K mutation produces a decreased catalytic turnover rate of the α_2_ NKA, not a complete loss-of-function [81], and it is unclear whether the expression of the α_2_ NKA is reduced in the brain of heterozygous E700K FHM2 mice [61].

The L263V FHM3 mutation produces gain-of-function of recombinant human Na_V_1.1 channels, mainly due to a reduction of channel inactivation and an increase in the persistent Na^+^ current component [82]. The L1649Q mutant human Na_V_1.1 channel, which was non-functional when expressed in a non-neuronal human cell line because of lack of plasma membrane delivery [82], showed an overall gain-of-function phenotype and could sustain high-frequency firing better than the WT channel (due to reduced frequency-dependent inactivation) when expressed in cortical interneurons or when the folding defects were partially rescued in the heterologous expression system [83]. This feature is shared by other FHM3 Na_V_1.1 mutants [49], and one can conclude that the gain-of-function modifications in gating (in particular inactivation) properties of recombinant human Na_V_1.1 channels produced by the L1649Q (and other FHM3 mutations) dominate over the loss-of-function modifications particularly during high frequency trains of action potentials. Accordingly, an increased persistent Na^+^ current and slower Na^+^ current inactivation kinetics were measured in Purkinje cells dissociated from homozygous L1649Q knockin mice and an increased Na^+^ current elicited by voltage ramps was measured in hippocampal GABAergic neurons in heterozygous L1649 mutants [60].

## The genetic mouse models of migraine share increased susceptibility to CSD

A key migraine-relevant phenotype that FHM1, FHM2 and FHM3 animal models as well as the MA/FASPs mouse model have in common is increased susceptibility to experimentally induced CSD. A lower stimulation threshold for CSD initiation in these migraine models was revealed in vivo by inducing CSD using either focal electrical stimulation in anesthetized [55–57, 60] or freely behaving mice [59], focal high K^+^ stimulation in anesthetized mice [50] or veratridine stimulation in awake head-fixed mice [15], or focal light stimulation in anesthetized [84] or freely behaving mice expressing channelorhodopsin2 (optogenetic CSD) [85]. A lower CSD stimulation threshold in the mutants was also found in vitro (using focal high K^+^ pulses stimulation) [37, 60, 62, 71, 86]. Finally, prolonged epidural high KCl application in vivo in anesthetized or awake head-restrained mutant mice elicited a higher frequency of CSDs than in WT mice [50, 60, 87, 88]. The rate of CSD propagation was increased in the FHM1 and FHM2 animal models, both in vivo and in vitro [55, 56] [37, 57, 61, 71, 84–88], but not in the FHM3 knockin mice in vivo [59, 60] and only as a trend in the FA/FASPS mouse model in vivo [50].

In agreement with the larger Ca_V_2.1 gain-of-function produced by the S218L compared to the R192Q mutation [55, 56, 68, 69], the strength of CSD facilitation as well as the severity of the post-CSD neurological motor deficits and the propensity of CSD to propagate into subcortical structures were larger in homozygous S218L compared to homozygous R192Q knockin mice [55, 56, 87, 89, 90]. Moreover, unlike the R192Q mice, the S218L mice frequently developed multiple CSDs after a single CSD-inducing stimulus, which were more frequent in homozygous compared to heterozygous mice [56, 85]. Homozygous S218L mice also showed spontaneous non-fatal and fatal seizures, resulting in reduced life expectancy [91]. Seizures, induced by electrical stimulation of sensorimotor cortex in freely-behaving mice, were followed by multiple CSDs and death in homozygous S218L mice (but not in heterozygous S218L mice). In anesthetized homozygous S218L mice, propagation of the spreading depolarizations to the brainstem was observed in association with fatal seizures; the appearance of seizure-related spreading depolarization in the brainstem correlated with respiratory arrest, followed by cardiac arrest and death, suggesting that brainstem spreading depolarization induces apnoea and causes death by suppression of brainstem respiratory control [91, 92]. Indeed, treatment with NMDAR antagonists prevented seizure-related brainstem spreading depolarization and apnoea as well as fatal outcome in freely behaving homozygous S218L mice [92]. These unique CSD features might contribute to the severe human clinical syndromes caused by the S218L mutation [63].

In agreement with the larger overall gain-of-of function of recombinant human Na_V_1.1 channels produced by the L263V compared to the L1649Q FHM3 mutation [82, 83], while heterozygous L1649Q knockin mice were fully viable [60], heterozygous L263V knockin mice had a substantially reduced lifespan (with peak mortality between P21-35, which may be related to a developmental peak in Na_V_1.1 expression in this age range [47]) [59, 93]. The premature mortality was due to spontaneous sudden apnoea preceded by a profound brainstem depolarization in the absence of epileptiform activity [93]. Interestingly, apparent life-­threatening apnoeic events were reported in an infant with the homozygous L263V missense mutation, which causes typical FHM3 in heterozygous family member (without epilepsy, respiratory dysfunctions, coma or death); this suggests that the brainstem phenotype of L263V mice is more severe than in patients [93]. In contrast with the seizure-related brainstem spreading depolarizations in homozygous S218L FHM1 mice, treatment with a potent NMDAR antagonist was ineffective at blocking brainstem DC shifts, apnoea or death in heterozygous L263V FHM3 mice, suggesting that a different mechanism may underlie the profound brainstem depolarization observed in L263V mice [93]. Interestingly, heterozygous L263V knockin mice receiving GS967, a preferential inhibitor of persistent Na^+^ current [94], showed improved survival, while the sudden death phenotype resumed after switching back to normal chow [93]. Homozygous L1649Q FHM3 knockin mice had a substantially reduced lifespan too, and systemic administration of GS967 considerably improved their survival [60]. GS967 rescued the increase in the persistent ramp Na^+^ currents recorded in hippocampal fast-spiking neurons in heterozygous L1649Q knockin mice [60], and rescued the gain-of-function properties of the recombinant L263V mutant human Na_V_1.1 channels [93], thus implicating the gain-of-function of the persistent Na^+^ current in the premature death.

A relatively large subset (38%) of heterozygous L263V FHM3 knockin mice with implanted electrodes in V1 and M1 (which further reduced their lifespan) showed frequent, apparently spontaneous, CSD events (almost 1 every hour, propagating in one hemisphere from V1 to M1). The frequency of spontaneous CSDs was much (4 times) lower in (a smaller subset of) freely behaving mice with epicranial electrodes [59], suggesting that some (or perhaps most) of the events may have been due to the mechanical stimulation or tissue damage caused by the implanted electrodes. A similar very low frequency of spontaneous CSDs was observed in a small subset of implanted homozygous R192Q and heterozygous S218L mice (which were first detected in either visual or motor cortex and sometimes simultaneously at both locations) [85]. Interestingly, all apparently spontaneous CSDs in FHM1 mice were observed during the light period (during mouse sleep) and the mice were predominantly in non-REM sleep during the 10 min preceding a spontaneous CSD (but shortly before CSD was first detected the mice woke up, in all except one case) [85]. Of note, the threshold for induction of optogenetic CSD in freely behaving WT mice is also lower during non-REM sleep compared to active states [85, 95].

## Functional alterations in the cerebral cortex of the genetic mouse models of migraine

We will focus on reviewing the functional studies in the cerebral cortex of FHM mouse models carrying mutations causing pure FHM and in the MA/FASPS mouse model. We will only briefly mention some of the functional studies in the FHM mutants carrying the severe syndrome-causing mutations, because these are likely less relevant for the understanding of mechanisms impacting most migraineurs.

### FHM1 mice

Measurement of synaptic transmission at different intracortical and thalamocortical (TC) synapses in acute brain slices from R192Q FHM1 knockin mice (FHM1 mice) invariably showed enhanced glutamatergic transmission at the excitatory synapses, due to enhanced action-potential (AP) evoked Ca^2+^ influx through mutant presynaptic Ca_V_2.1 channels, and enhanced probability of glutamate release [71, 96, 97]. The excitatory synapses in the primary somatosensory (S1) cortex analysed in FHM1 mice included synapses onto principal neurons (PCs) (L2/3 PC-PC synapses and TC neuron-L4 PC synapses) and excitatory synapses onto inhibitory interneurons (INs) (L2/3 PC-fast spiking (FS) IN and L2/3 PC-somatostatin expressing (SOM) IN synapses as well as TC neuron-L4 FS IN synapses). While the strength of excitatory transmission was similarly enhanced at all these synapses, their synaptic short-term plasticity properties (elicited by 10–25 Hz action potential trains) were differentially affected by the FHM1 mutation. The short-term depression (STD) at intracortical L2/3 PC-PC and PC-FS IN synapses and at thalamocortical synapses onto L4 PCs was enhanced in FHM1 mice, but STD was unaltered at thalamocortical synapses onto FS INs, and also unaltered was the short-term facilitation at L2/3 PC-SOM IN synapses [71, 96, 97]. Despite the similar gain-of-function of synaptic transmission, the short-term plasticity at different excitatory synapses may be differently affected by the FHM1 mutation depending on their specific plasticity mechanisms, e.g. on whether and to what extent these mechanisms depend on initial release probability or on AP-evoked calcium influx [98, 99].

In striking contrast with glutamatergic transmission, GABAergic transmission at different cortical inhibitory synapses was unaltered in FHM1 mice, despite being initiated by Ca_V_2.1 channels [71, 76, 97]. The lack of effect on cortical inhibitory synaptic transmission in contrast with the gain-of-function effect on excitatory transmission is likely a common feature of FHM1 mutations since it was shown also for the S218L mutation [75]. In light of the findings of Vecchia et al. [76], including unaltered Ca_V_2.1 current in cortical multipolar (FS and non-SOM) INs from FHM1 mice, a likely mechanism underlying the unaltered cortical inhibitory neurotransmission in FHM1 mice is the expression of interneuron-specific Ca_V_2.1 channels whose gating is not affected by the FHM1 mutation. While the molecular mechanisms underlying the differential effect of FHM1 mutations on the Ca_V_2.1 channels expressed in excitatory and inhibitory cortical neurons remain unknown, possible mechanisms include the expression of different channel splicing variants and/or different auxiliary subunits or different regulatory proteins at the synaptic active zones. An important general implication is that neuron subtype-specific and synapse-specific effects may help to explain why a mutant calcium channel that is widely expressed in the nervous system produces the specific brain dysfunctions underlying FHM.

As a whole, the analysis of cortical synaptic transmission in FHM1 mice supports the general conclusion that FHM1 mutations enhance excitatory synaptic transmission without directly affecting inhibitory synaptic transmission, regardless of the identity of the presynaptic and the postsynaptic target neuron, although the latter may determine how the mutations affect short-term plasticity at the excitatory synapses.

Note that an enhancement of excitatory synaptic transmission with unaltered inhibitory synaptic transmission does not necessarily lead to network hyperexcitability and increased firing rates because, while the enhanced excitatory transmission at the intracortical synapses between PCs (and/or at the afferent synapses on PCs in long range connections) may certainly increase network excitation, the enhanced excitatory transmission at the intracortical synapses between PCs and INs (or at the afferent synapses onto INs) may lead to increased recruitment of INs which may increase network inhibition. Indeed, both the thalamocortical disynaptic feedforward inhibition mediated by FS INs in L4 PCs and the frequency-dependent disynaptic feedback-lateral inhibition mediated by SOM INs in L2/3 PCs were enhanced in FHM1 mice, as a consequence of the enhanced glutamatergic transmission onto the INs [96, 97]. There is evidence that, in FHM1 mice, this increased inhibition may efficiently counteract the increase in recurrent excitation, which one would predict from the enhanced excitatory transmission onto PCs, and may even prevail in certain conditions. In fact, as a consequence of the differential effect of the FHM1 mutation on short-term synaptic plasticity at the TC synapses on L4 PCs and FS neurons, the excitatory–inhibitory (E/I) balance set by the TC feedforward inhibition microcircuit in L4 PCs of the somatosensory cortex, was relatively skewed toward inhibition during repetitive thalamic stimulation in FHM1 mice [96]. As a consequence of this (and probably also of the increased intracortical feedback inhibition) the cortical recurrent network activity induced in thalamocortical slices by repetitive thalamic firing was reduced in FHM1 compared to WT mice [96].

There is also in vivo evidence that a net inhibitory circuit output can arise from a mutation that increases excitation at the synaptic level in FHM1 mice. Recordings of visual evoked potentials and multiunit firing activity (MUA) in L4 of the primary visual cortex in response to visual grating reversal in awake head-fixed FHM1 mice revealed a lower MUA peak amplitude at high contrast in FHM1 compared to WT mice, a finding consistent with a shift of the E/I balance in L4 PCs towards inhibition [100]. Both the decreased MUA amplitude and the decreased E/I ratio could be simulated using a simple V1 spike network by introducing the intracortical and thalamocortical synaptic alterations found in the FHM1 mouse model described above [100]. The same model with the same FHM1 synaptic alterations could also simulate the larger increase in high γ band (70–100 Hz) synchronization (and smaller increase in the β-low γ band, 12–40 Hz) revealed by spectral analysis of the local field potential responses to the high contrast visual stimulus in FHM1 compared to WT mice [100]. Perhaps consistent with these data, a previous study measuring in anesthetized FHM1 mice the neuronal Ca^2+^ changes elicited by whisker stimulation in the somatosensory barrel cortex, revealed smaller Ca^2+^ responses in FHM1 compared to WT mice [101]. However, in freely behaving FHM1 mice, the initial increase in MUA activity in visual cortex in response to single-flash blue light pulses was unaltered, and the MUA suppression following the initial MUA increase was reduced [102]. Moreover, the EEG responses to flash light stimulation at different frequencies revealed enhancement of photic drive for stimulation frequencies in the β-low γ band in freely behaving FHM1 mice, which were able to follow the “chirp” frequency stimulation up to 40 Hz, compared to a maximum of 25 Hz in WT mice [102]. Power spectra of intracortical local field potential recordings in V1 and M1 during baseline spontaneous activity in freely behaving mice revealed an increased power in the low γ range (30–45 Hz, high γ band not analysed) in FHM1 compared to WT mice in both active wake states and REM and non-REM sleeping states [85].

Taken together, these studies give us the lesson (relevant to any migraine model, not just FHM1) that the circuit effects of a mutation cannot be trivially predicted from its synaptic/cellular effects. Excitable conditions (e.g. favoring CSD) might be present in one circuit, while opposite effects prevail in another. Moreover, they might be dependent on brain/behavioural state.

### FHM2 mice

Heterozygous W887R FHM2 knockin mice (FHM2 mice) show a reduced rate of clearance of synaptic glutamate, released during neuronal activity, in both acute cortical slices in response to extracellular stimulation and in vivo in response to whisker stimulation in awake head-fixed animals [15, 37, 40, 86]. The density of GLT-1 glutamate transporters, which mediate the majority of glutamate clearance in the adult murine neocortex [103], was reduced 50% at cortical perisynaptic astrocytic processes in FHM2 mice, a reduction that mirrors the 50% reduction in expression of the α_2_ NKA, pointing to the reduced expression of astrocytic glutamate transporters as the main mechanism underlying the reduced rate of glutamate clearance in FHM2 mice [37]. Interestingly, the relative impairment of glutamate clearance in these mice was activity dependent, being larger after a train of pulses than after a single pulse stimulation and increasing with increasing stimulation frequency in cortical slices [37].

Imaging extracellular glutamate using two-photon microscopy in awake FHM2 mice revealed the presence of spontaneous focal, high-amplitude glutamate fluorescence events, that were called glutamate plumes and were shown to arise as a consequence of impaired astrocyte clearance of glutamate released from neuronal synapses [15]. Glutamate plumes were observed predominantly in layer 1 (L1), a layer that is characterized by reduced coverage of glutamatergic synapses by perisynaptic astrocyte processes expressing GLT-1a and hence by a reduced glutamate clearance capability in comparison with deeper layers [15]. Thus, glutamate plumes may be markers of inefficient glutamate clearance. Spontaneous glutamate plumes were only very rarely observed in WT mice, but could be induced in WT cortex by either pharmacological inhibition of glutamate transporters or by strong depolarizing stimuli, like those used to induce CSD [15].

As a result of the slowing of glutamate clearance and the consequent increased glutamate spillover, the glutamate NMDA receptor (NMDAR) excitatory postsynaptic current elicited in L2/3 pyramidal cells by stimulation of L1 neuronal afferents had a larger amplitude and a slower decay in FHM2 compared to WT mice, due to activation of extrasynaptic diheteromeric GluN1-N2B NMDARs [86]. This is in line with the proposed role for extrasynaptic GluN1-N2B NMDARs as primary detectors of glutamate spillover [104, 105], and it is also consistent with the higher affinity for glutamate (and lower susceptibility to Mg^++^ block) of GluN1-N2B compared to other NMDA receptors, including the triheteromeric GluN1-N2A-N2B receptors [106, 107]. The relative increase of NMDAR activation in FHM2 mice was activity-dependent, in the sense that it was larger after a high-frequency train of pulses than after single pulse stimulation [86]. Interestingly, the generation of long-lasting NMDA spikes in the tuft dendrites of layer 5 pyramidal cells (involving activation of extrasynaptic NMDARs by glutamate spillover [108, 109]), was facilitated in FHM2 mice [40]. As a consequence, the output somatic burst firing promoted by NMDA spikes was increased in FHM2 compared to WT mice [40].

In addition to the rate of glutamate clearance, the rate of clearance of K^+^ ions released during neuronal activity was also reduced in FHM2 mice [37] [40], showing the important role of the α_2_ NKA in both glutamate and K^+^ clearance [41]. Restoring the reduced α_2_ NKA expression level in FHM2 mice to the WT level (via viral delivery of *Atp1a2* to cortical astrocytes) rescued the reduced glutamate and K^+^ clearance as well as the facilitation of the dendritic NMDA spikes and the increased output firing induced by these long-lasting spikes in FHM2 mice [40].

### FHM3 mice

Measurement of intrinsic firing in different types of neurons in acute cortical slices from heterozygous L1649 FHM3 knockin mice (FHM3 mice) revealed an increased action potential frequency in L4 FS INs but an unaltered frequency in regular spiking INs and in layer 5 pyramidal cells [60]. The finding that the frequency of spontaneous inhibitory postsynaptic currents in cortical pyramidal cells was enhanced in FHM3 mice provided evidence that the spontaneous activity of cortical INs is increased in cortical slices from FHM3 mice [60]. In contrast miniature inhibitory postsynaptic currents were unaltered*.* Spontaneous excitatory postsynaptic currents were not measured.

The preferential inhibitor of the persistent Na^+^ current GS967 reduced the AP frequency in FS INs in both WT and FHM3 mice, but, interestingly, the effect of GS967 was larger in WT INs at high frequency [60], suggesting that the hyperexcitability of FS INs in FHM3 mice may not be *directly* due to gain-of-function of the persistent Na^+^ current. Indeed, simulations of neuronal firing properties showed that an enhanced persistent Na^+^ current induces just small modifications of the INs firing frequency [110]. However, increased persistent Na^+^ current results in increased K^+^ and Na^+^ fluxes at each action potential and a large increase in the K^+^ charge needed to repolarize the action potential, predicting an increase of the extracellular K^+^ concentration and an important energetic burden on firing neurons with increased persistent Na^+^ current [94, 110].

### CK1δ_T44A_ MA/FASPS mice

Measurement of excitatory and disynaptic inhibitory postsynaptic currents evoked in L2/3 PCs by stimulation of L4 PCs (as well as of spontaneous synaptic currents in L2/3 PCs) in S1 cortical slices from CK1δ_T44A_ mice revealed that neither phasic excitatory nor inhibitory synaptic transmission was altered in these MA mouse models in response to transient stimulation [62]. However, measurement of excitatory postsynaptic currents evoked by trains of stimuli at different frequencies revealed a frequency-dependent reduction of short-term depression (STD) at the L4 PC-L2/3 PC synapses (consistent with reduced sensory adaptation) in CK1δ_T44A_ mice. This was particularly evident with high frequency (50 Hz) presynaptic stimulation [62]. The mechanism of this STD deficit was a calcium-dependent enhancement of the size of the readily releasable pool of synaptic vesicles in the presynaptic terminals of CK1δ_T44A_ mice. In striking contrast, STD of the *inhibitory* postsynaptic currents evoked by train of stimuli at different frequencies was unaltered in CK1δ_T44A_ mice [62]. As a result of the STD deficit at the excitatory synapses, glutamate release in response to high-frequency (50 Hz) stimulation was increased in the MA mouse model (as revealed by imaging glutamate transients in L2/3) [62]. At a local circuit level, long duration action potential bursts elicited in L2/3 pyramidal cells triggered an increase in recurrent excitation in CK1δ_T44A_ compared to WT cortical slices, and at the network level, anesthetized CK1δ_T44A_ mice showed a longer duration of ‘up state’ activity [62].

## Mechanisms underlying facilitation of CSD initiation in the genetic mouse models of migraine

CSD rescue experiments have shown that there is a causative link between increased Ca_V_2.1-dependent glutamatergic transmission at cortical synapses and facilitation of experimental CSD in FHM1 mice. The facilitation of initiation and propagation of CSD (induced by high KCl puffs) in cortical slices from FHM1 mice was completely eliminated when AP-evoked glutamate release at cortical synapses was brought back to WT values by partially inhibiting Ca_V_2.1 channels [71]. Besides uncovering a key mechanism underlying CSD susceptibility in FHM1, these findings point to a role of Ca_V_2.1-dependent glutamate release in CSD initiation. Such role is also supported (in the inverse) by the earlier in vivo finding of an increased stimulation threshold for CSD induction (by focal electrical stimulation) in mutant mice carrying mutations which cause partial loss-of-function of Ca_V_2.1 channels and reduced K^+^-evoked glutamate rise in neocortex [111], as well as by the recent pharmacological evidence (obtained by imaging the intrinsic optic signal at the CSD initiation site in cortical slices) that blocking Ca_V_2.1 channels increases more than fourfold the CSD initiation threshold and prevents CSD propagation in WT mice [112]. This major role of Ca_V_2.1 channels for CSD initiation contrasts with the minor role played by the other voltage-gated Ca^2+^ channels [113] and correlates with the dominant role of Ca_V_2.1 channels in controlling glutamate release at cortical synapses [29, 114, 115].

The important role of excessive glutamatergic transmission in CSD susceptibility is underscored and supported by functional studies in both FHM2 and CK1δ mutant mice. Capuani et al. [37] showed that the defective glutamate clearance at cortical excitatory synapses can account for most of the facilitation of CSD initiation and for a large part of the facilitation of CSD propagation uncovered in cortical slices from FHM2 mice. The reduced rate of K^+^ clearance likely accounts for the remaining unaccounted CSD facilitation [37]. Confirming the key role of impaired glutamate clearance by GLT-1 transporters in facilitation of CSD initiation and propagation in FHM2 mice, GLT-1 knockout mice with about 70% reduction of GLT-1 expression in the brain show increased susceptibility to experimentally induced CSD and increased rate of CSD propagation [116]. In contrast, CSD induction and propagation were not altered in mice with glutamate transporters GLAST and EAAC1 knocked out [116].

Imaging of extracellular glutamate at the site of CSD initiation (by veratridine) in awake head-fixed mice revealed that an increase in basal glutamate and in frequency of glutamate plumes precedes and may predict CSD initiation in both FHM2 and WT mice [15]. Blocking voltage-gated Ca^2+^ channels with Ni^2+^ eliminated the glutamate rise preceding CSD, inhibited the incidence of plumes and prevented CSD initiation by threshold stimulation in most FHM2 mice [15]. Interestingly, the levels of both glutamate and plumes frequency just prior to initiation of CSD by a threshold stimulation were quite similar in WT and FHM2 mice, despite the fact that the threshold stimulation for CSD initiation was lower in FHM2 mice [15]. These findings support the idea of a threshold level of glutamate necessary for CSD ignition regardless of genotype. Moreover, if glutamate plumes (in L1) are a marker of inefficient glutamate clearance, then these findings may suggest that a critical threshold level of impairment of glutamate clearance (due to decreased cycling rate of glutamate transporters consequent to stimulus-induced depolarization, extracellular K^+^ increase, intracellular accumulation of Na^+^ and glutamate) is necessary for CSD initiation regardless of genotype. Due to their reduced rate of glutamate clearance, this threshold level is reached with a stimulus of lower intensity and more rapidly in FHM2 mice, thus accounting for the facilitation of CSD initiation.

Finally, lowering the extracellular [Ca^2+^] eliminated both the difference in STD at L4-L2/3 synapses of WT and CK1δ_T44A_ mice and the genotype difference in stimulation threshold for CSD initiation in cortical slices [62]. Thus, the findings of Suryavanshi et al. [62] support the conclusion that a frequency-dependent presynaptic gain of function at glutamatergic synapses accounts for the facilitation of CSD initiation in the MA mouse model. They also underscore a potentially common role of [Ca^2+^]-dependent presynaptic excitability across several genetic models (FHM1, FHM2, MA).

**Fig. 1:**
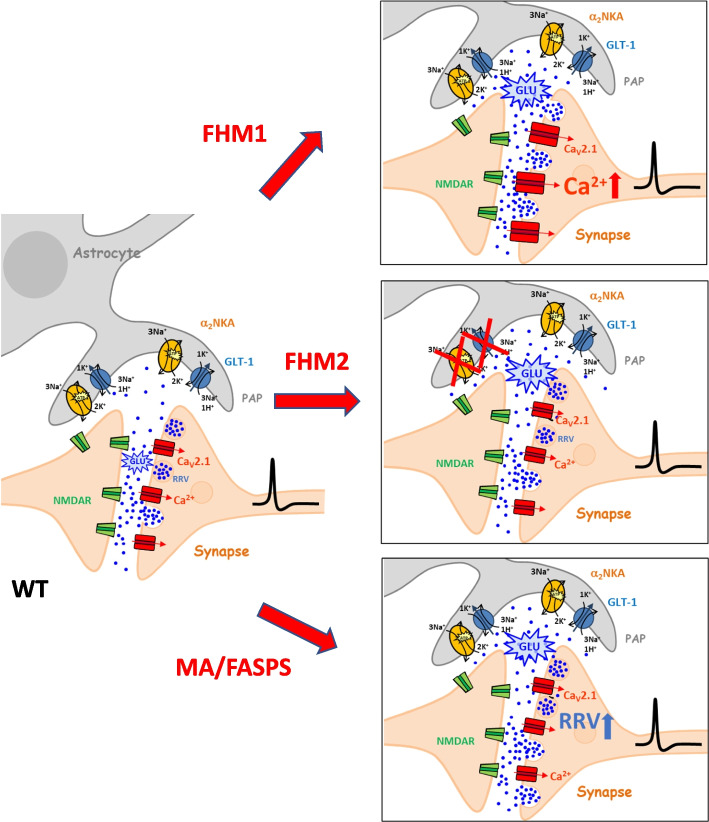
FHM1, FHM2 and MA/FASPS mice share enhanced glutamatergic neurotransmission at cortical synapses. Different mechanisms underlie the enhanced cortical glutamatergic transmission in the three genetic mouse models of migraine: enhanced glutamate release due to enhanced Ca^2+^ influx through presynaptic Ca_V_2.1 channels in FHM1; reduced rate of glutamate clearance due to reduced density of GLT-1 glutamate transporters and α_2_ NKAs in perisynaptic astrocytic processes in FHM2; Ca^2+^-dependent enhancement of readily releasable vesicles (RRV) at the active zones in MA/FASPS

Overall the findings in the FHM1, FHM2, and MA mouse models support the conclusion that an excessive cortical glutamatergic transmission, due either to increased glutamate release or impaired glutamate clearance (Fig. 1), underlies the facilitation of CSD initiation in these genetic mouse models of migraine.

**Fig. 2:**
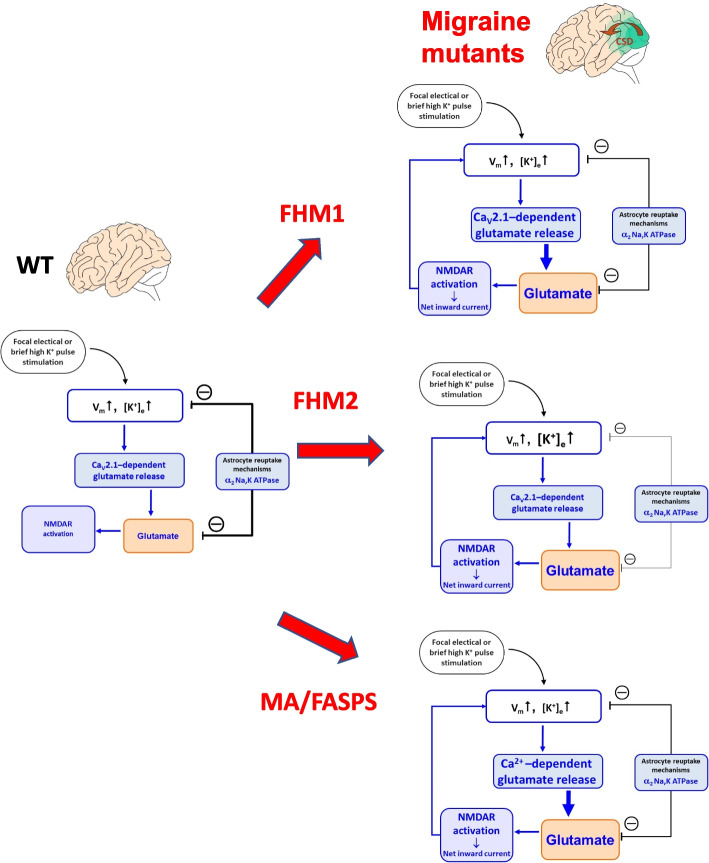
Excessive cortical glutamatergic neurotransmission underlies the facilitation of CSD initiation in FHM1, FHM2 and MA/FASPS mice. CSD is not induced in WT mice by the threshold depolarizing stimulus that initiates CSD in FHM1, FHM2 and MA/FASPS mice, because only in the mutants this stimulus increases glutamate above the threshold level which leads to cooperative activation of synaptic and extrasynaptic NMDARs above the critical level, that generates a net self-sustaining inward current and initiates the positive feedback cycle that is necessary to ignite a CSD. The enhanced susceptibility to CSD is due to enhanced glutamate release in FHM1 and MA/FASPS and to impaired rate of glutamate clearance in FHM2

As indicated by glutamate imaging at the site of CSD initiation in FHM2 and WT mice, a critical threshold level of glutamate is necessary for CSD ignition regardless of genotype. This implicates glutamate, rather than K^+^, as the ‘switch’ that takes the tissue over CSD threshold level (more below). As shown in the FHM2 model, this threshold level (which appears similar in WT and mutant animals) is likely reached with a stimulus of lower intensity and more rapidly in the migraine mouse models, thus accounting for the facilitation of CSD initiation (Fig. 2).

But why and how does this critical threshold level of glutamate initiate CSD regardless of genotype? Several lines of evidence point to the activation of a critical threshold level of glutamate NMDARs as necessary for initiation of CSD in both WT mice and the FHM models (regardless of genotype).

The involvement of NMDARs in CSD initiation in the cerebral cortex of WT animals is supported by many in vivo and in vitro pharmacological studies using electrical or high K^+^ focal stimulation to induce CSD (see [3] for a review and references) and also, more recently, by in vivo studies using focal light stimulation to induce optogenetic CSD in anesthetized [117] or awake head-fixed mice [118]. It is important to note that in this discussion, we are focusing on conditions relevant to migraine; i.e. uninjured brain, with normal metabolic parameters (perfusion, oxygen and glucose levels), and focal rather than diffuse stimulation. The mechanisms of initiation of spreading depolarizations in metabolically compromised tissue and with diffuse stimulation are different, and in particular the activation of NMDARs and Ca_V_-dependent glutamate release appears to play a relatively minor role in these conditions ( [3, 119] and references therein).

Considering the studies using focal stimulation in WT normally metabolizing cortical tissue (the condition of all the studies in the migraine genetic mouse models reviewed above), particularly relevant are the CSD thresholding investigations in which the depolarizing stimulus was varied from subthreshold values up to CSD threshold (or higher) in the presence and absence of NMDAR antagonists. The necessity of NMDAR activation for CSD initiation at threshold stimulation (or not much higher) was clearly shown by finding that partial inhibition of NMDARs (by subsaturating doses of specific antagonists) led to an increase in the stimulation threshold for CSD induction (e.g. [120]), and by finding that complete inhibition of NMDARs (by saturating doses of the antagonists) blocked CSD measured at or very close to the site of stimulation in vivo (e.g. [117, 118, 121] at threshold, 3 and 1.5 times threshold stimulation, respectively). Strong evidence that a critical threshold level of NMDAR activation is necessary for CSD initiation at threshold stimulation and that, if NMDARs are blocked, even largely suprathreshold stimuli are unable to initiate CSD, was recently provided by a study in which the intrinsic optic signal at the site of CSD initiation and the membrane potential of a pyramidal neuron located very close to this site were simultaneously measured in WT cortical slices in response to brief KCl puffs of increasing duration up to the threshold duration eliciting a CSD and then up to 10–15 times this threshold [112]. This study showed that CSD initiation is not determined by the level of extracellular K^+^ or neuronal depolarization per se (as it is often assumed), but requires the Ca^2+^ channel (mainly Ca_V_2.1)-dependent activation of a threshold level of NMDARs. Interestingly, the onset of CSD occurred with a delay of several seconds relative to the rapid strong neuronal depolarization produced by the threshold KCl puff, and the CSD delay coincided with the delay necessary to reach the threshold level of NMDAR activation [112]. It is interesting to note that optogenetic CSD in anesthetized mice also started with a delay of up to 10–20 s after the end of the illumination and the associated negative field potential [117].

In awake head-fixed WT mice, the beginning of the slow rise of extracellular glutamate and of the increase in glutamatergic plume frequency (a likely marker of inefficient glutamate clearance) at the site of CSD initiation by a threshold KCl concentration occurred a few seconds before CSD onset [15], a time similar to the delay necessary to reach the threshold level of NMDAR activation measured in slices [112]. This suggested the hypothesis that this delay might correspond to the time necessary to raise glutamate above the critical threshold level necessary for CSD initiation, and this occurs as a consequence of a critical level of impairment of glutamate clearance (potentially due to decreased cycling rate of glutamate transporters consequent to the depolarization and the extracellular K^+^ increase and the intracellular accumulation of Na^+^ and glutamate produced by the stimulus) [112]. Consistent with this hypothesis is the finding that the rise of extracellular glutamate to the threshold level necessary for CSD initiation was faster in FHM2 compared to WT mice [15].

Investigation of the initiation mechanisms of CSD in FHM1 mice showed that a delayed activation of NMDARs above a critical threshold is necessary for initiation of CSD also in these mutants. Importantly, this threshold level was quantitatively similar in FHM1 and WT mice (similar to what was observed in FHM2 and WT mice for the glutamate threshold level), but it was reached with a stimulus of lower intensity and more rapidly in FHM1 compared to WT mice, thus accounting for the facilitation of CSD initiation (Vitale et al. 2024, unpublished findings, presented at the FENS 2024 meeting https://www.world-wide.org/fens-24/mechanisms-facilitation-cortical-spreading-c73f4f51/qr_code.png).

Specific inhibition of di-heteromeric GluN1-N2B NMDARs in FHM2 mice increased the CSD threshold and reduced the CSD velocity to values close to those measured in WT mice, suggesting that the facilitation of CSD initiation in FHM2 mice is mainly due to activation of this subtype of NMDARs, which appears to be mainly located at extrasynaptic sites [86].

Overall the findings in the FHM1, FHM2, and MA mice suggest a model of CSD initiation in which a threshold depolarizing stimulus releases enough glutamate to overwhelm the reuptake capacity of the (mainly astrocytic) glutamate transporters, thus leading to cooperative activation of (synaptic and extrasynaptic) NMDARs above the critical level necessary to (generate a net self-sustaining inward current and thus) initiate the positive feedback cycle that ignites a CSD [16]. Increased susceptibility to CSD in the migraine mouse models is due to the fact that the critical glutamate and NMDAR activation thresholds are reached with stimuli of lower intensity as a consequence of the functional alterations produced by the migraine mutations we have discussed (Fig. 2).

What about the mechanisms underlying facilitation of CSD initiation in FHM3 mice? The preferential inhibitor of the persistent Na^+^ current GS967 reduced the success rate of CSD induction by focal high KCl in acute cortical slices from both WT and FHM3 mice, but (in contrast to its effect on FS INs action potential firing; see above) the effect of GS967 was relatively larger in FHM3 mice and, as a consequence, the difference in CSD induction rate between the two genotypes was eliminated [60]. This finding supports the conclusion that the persistent Na^+^ current contributes to determine the CSD threshold and is involved in CSD initiation in WT mice and that the increase in persistent Na^+^ current carried by Na_V_1.1 channels in FHM3 mice can account for the CSD facilitation in the FHM3 mutants.

The mechanism by which an increased Na_V_1.1 persistent current in FS interneurons and an increased firing rate of these neurons facilitate CSD initiation is not obvious. Simulations, using a two-neuron conductance-based model of interconnected GABAergic and pyramidal glutamatergic neurons with incorporated ionic concentration dynamics, suggest that the pathophysiological link between increased persistent Na^+^ current in the interneurons and increased susceptibility to CSD might be the larger accumulation of extracellular K^+^ at each interneuron action potential [94] with consequent build-up of [K^+^]_e_ and increased firing of excitatory neurons [110]. Measurement of [K^+^]_e_ far from the CSD initiation site revealed an earlier rise of [K^+^]_e_ preceding the propagating CSD front and a larger [K^+^]_e_ at the point of maximal rate of [K^+^]_e_ rise during the CSD in FHM3 compared to WT mice [60]. The increase and early shift in [K^+^]_e_ were measured 1 mm away from the CSD initiation site, which is a large distance relative to typical foci of CSD induction, and hence seems more relevant for CSD *propagation* than CSD initiation. However this finding was considered to support the hypothesis that a larger build-up of extracellular K^+^ underlies the facilitation of CSD initiation in FHM3 mice [60, 94, 110]. This is tenable if the mechanisms of propagation and initiation are similar. Since an increased [K^+^]_e_ would lead to an increase of both AP-dependent and AP-independent glutamate release, this hypothesis could actually be consistent with the key role of a threshold level of glutamate and NMDARs in CSD initiation (supported by the studies in the FHM1, FHM2 and MA mouse models discussed above).

However, we would like to suggest that an increased persistent Na^+^ current in cortical pyramidal cells might be an additional mechanism that could contribute to facilitation of CSD initiation in FHM3 mice. Na_V_1.1 channels are also expressed in excitatory cortical neurons, particularly in the upper cortical layers (where, in contrast with the lower layers, the expression does not seem much lower than in interneurons) [122] and the persistent Na^+^ current in CA1 pyramidal cells was shown to greatly increase when [K^+^]_e_ was raised to 10–20 mM [123]. In modeling studies aiming to define the minimal biophysical machinery capable of initiating CSD [16, 124] either a persistent Na^+^ current or an NMDAR current in pyramidal cell apical dendrites could generate the net self-sustaining inward current that initiates the positive-feedback cycle igniting CSD. When both currents were present the CSD threshold was lower and the onset faster.

## Insights into mechanisms of initiation of spontaneous CSDs in migraine from the genetic mouse models

A key insight from the genetic models is the role of glutamatergic transmission as the likely ‘tipping agent’ or ‘switch’ that mediates the all-or-none transition of the network to CSD. K^+^ elevations might set the stage, but a surge of glutamate, whether due to increased release or impaired reuptake, appears necessary to cross the CSD initiation threshold. This occurs when the glutamate surge leads to activation of a critical level of NMDARs and hence to generation of a net self-sustaining inward current that makes the neuronal depolarization and K^+^ elevations self-regenerative. Of course, it must be emphasized that there is a reciprocal relationship between K^+^ and glutamate that is also likely essential to CSD ignition: glutamate binding to post-synaptic receptors results in K^+^ release (particularly through NMDARs: [125, 126]), and extracellular K^+^ further depolarizes the postsynaptic membrane as well as, among other things, the presynaptic terminals responsible for glutamate release [126]. It seems unlikely that local conditions can be made propitious for initiation of CSD, especially in the uninjured migraine brain, without the amplificatory mechanisms enabled by this reciprocal relationship.

Glutamate levels are normally tightly governed by reuptake mechanisms, primarily driven by astrocytic glutamate transporters whose action is powered by the ionic gradients and the membrane polarization generated by the Na^+^/K^+^ ATPase [41, 103, 127, 128]. A saturation of reuptake due to overwhelming synaptic release (e.g. in FHM1, MA), or an impairment of reuptake itself (e.g. FHM2) appears necessary to achieve a focus of CSD ignition. Note also the potential/likely role of elevations of extracellular K^+^: by depolarizing (in this case) astrocytic membranes and decreasing the K^ +^ electrochemical gradient, K^+^_e_ decreases the efficiency of glutamate reuptake [103, 127, 128], favoring an amplificatory cycle of glutamate/K^+^ release that results in CSD-threshold levels of glutamate. Finally, another potential amplifying mechanism that could likely contribute to CSD ignition involves the reduced perfusion due to constriction of arterioles above ~ 15–20 mM [K^+^]_e_ [21, 22] (Box 1).

We thus have an approximate understanding of at least some of the variables involved at the tipping point of CSD induction. But what brings an uninjured cortex into the already physiologically tenuous state where this tipping point is enabled? The genetic mouse models of migraine support the view of migraine as a disorder of the brain characterized by dysfunctional regulation of the E/I balance in neuronal circuits [7, 129], and suggest the hypothesis that, in certain conditions, an altered E/I balance in specific cortical circuits may bring them to the tipping point [130]. But what are the relevant cortical circuits and what are the specific conditions in which this may occur?

CSD is most easily inducible in the upper dendritic layers of neocortex [2, 3, 131]. These layers – 1 and 2/3—receive both robust cortical and neuromodulatory as well as thalamic excitatory afferents, and they are also subject to both inhibition and disinhibition due to canonical circuit motifs [132, 133]. Dendritic arbors are richly decorated with the NMDA receptors that are important to CSD ignition. If one layers dynamics onto the dendrites of a sensory cortical microcircuit, in the awake subject there is a continual barrage of incoming ‘bottom-up’ excitatory input (primarily from the thalamus), along with just as robust ‘top-down’ excitatory activity from higher sensory cortical regions, association cortex, executive regions, and salience network regions. This activity is both all-or-none (vesicular release at the synapse) and oscillatory (gamma oscillations and up-down states locally; theta and delta oscillations at larger distances). Affecting the tone of these dynamics is neuromodulatory activity, which can be both diffuse and quite precise (laminar specific). Finally, there are longer-term circuit modes such as different states of wakefulness, and different sleep stages. There is thus a host of circuit variables – from individual ionic conductances, to perisynaptic reuptake mechanisms, to synaptic and circuit architecture, to local, regional and global activity patterns – that could favor or disfavor entering a state where CSD ignition is possible. Onto this we need to layer the homeostatic factors – perfusion, oxygenation, ATP availability – that are arguably as important as the circuit variables in the genesis of CSD. Some metabolic compromise, brought about through reduced energy substrates (fasting), decreased perfusion (local thrombosis, local vasospam, dehydration), genetic mutations, or other causes, likely play a relevant role in favouring CSD ignition in migraine. The ‘metabolic’ and ‘excitation/inhibition’ factors are of course intimately intertwined. Finally, we need to account for the fact that, even in monogenic migraine models, migraine with aura, and thus CSD, is *episodic –* it is a low likelihood event – most of the time, it does *not* occur.

Thus far, relatively few investigations of sensory cortical microcircuits and of network activity in different states (at baseline and in response to sensory stimulation) have been performed in genetic models of migraine. It is difficult to envision how the circuit alterations revealed by these studies (see section “Functional alterations in the cerebral cortex”) may favour initiation of spontaneous CSDs. Possibly, the relevant microcircuits were not studied and/or they were not studied in the relevant conditions of neuromodulation or in the relevant brain state. It is quite interesting, in this respect, that in both WT and FHM1 mice, CSD susceptibility was increased in non-REM sleep [85, 95]. Given the episodic nature of migraine and of spontaneous CSDs, it seems important to study the circuit alterations in the genetic mouse models in the presence of typical migraine triggers.

The episodic nature of CSD and migraine, combined with the significant change from normal physiology that even pre-CSD conditions entail, lead us to hypothesize that *layers of triggering factors* need to be invoked to generate CSD in an uninjured brain. These layered triggering factors might occur in different combinations, with different intensities, during different migraine episodes. In Box 2, we propose a purely hypothetical example of how a mutation might interact with circuits and the environment to generate a ‘spontaneous’ CSD in a migraine subject.

### Box 2. A potential path from mutation to spontaneous aura

As a completely hypothetical example (relevant variables in parentheses), one might envision a female carrier of the CK1d mutation (genetic history, sex), who is mildly sleep and calorie deprived and dehydrated due to work stress (depleted energy substrates, ‘stressed’ circuit activity). She has a consistent pattern of visual auras (genetic excitability, experience dependent plasticity, possible circuit architecture changes). She is reading a report. A car passes by on the street below, reflecting sunlight into her eyes. In the dendritic layers of her V1 cortex, a subset of synapses has been responding to repetitive stimulation for many hours. The local cortical columns are minimally hypoperfused (dehydration) and neurons, astrocytes and perfusing arterioles are mildly energy depleted (ATP expenditure). In a focal region, perhaps 300 μm in diameter, the repetitive synaptic activity is associated with a progressive failure of adaptation, due to the increased size of the readily releasable pool of vesicles. Glutamate accumulates in 100–1000 synaptic clefts and the perisynaptic region, cooperatively activating synaptic and extrasynaptic NMDA receptors, depolarizing the dendritic membranes. K^+^ is released and like the glutamate, diffuses locally, causing depolarization of adjacent membranes (astrocytic, presynaptic neuronal, and vascular). Assuming continued afferent activity, more glutamate is released, and accumulates, resulting in more K^+^ release and depolarization. At first, compensatory changes occur: local parvalbumin interneurons fire, releasing GABA and generating inhibitory postsynaptic potentials in the dendrites to counter the excitatory barrage. Local arterioles dilate as [K^+^]_e_ rises from the normal range of 3.5 mM to 6–8 mM. But the specular reflection from the car window, while the woman is reading a particularly difficult part of the report requiring special attention, tips the balance. A larger than normal number of V1 synapses fire, layering onto the excitatory barrage in the depleted region. Regenerative glutamate/K^+^ release leads to [K^+^]_e_ in the 12–20 mM range. Disinhibitory circuits are activated and inhibitory transmission fails. Penetrating arterioles constrict rather than dilate because of their biphasic response to elevated [K^+^]_e_. Astrocytic glutamate reuptake is inhibited by membrane depolarization, increased [K]_e_ and accumulation of intracellular Na^+^ and glutamate. Finally, a critical mass of glutamate binds NMDA receptors and the net neuronal current becomes inward. A confluent focal region depolarizes at once, and a regenerative wave of glutamate and K^+^ release sets off a wave of CSD and our subject’s migraine aura.

## Data Availability

No datasets were generated or analysed during the current study.
